# An Update on Hepatocellular Carcinoma in Chronic Kidney Disease

**DOI:** 10.3390/cancers13143617

**Published:** 2021-07-20

**Authors:** Fabrizio Fabrizi, Roberta Cerutti, Carlo M. Alfieri, Ezequiel Ridruejo

**Affiliations:** 1 Division of Nephrology, Dialysis and Transplantation, Ca’ Granda IRCCS Foundation and Maggiore Policlinico Hospital, 20122 Milano, Italy; roberta.cerutti@policlinico.mi.it (R.C.); carlo.alfieri@policlinico.mi.it (C.M.A.); 2 Department of Clinical Sciences and Community Health, University of Milan, 20122 Milan, Italy; 3 Hepatology Section, Department of Medicine, Centro de Educación Médica e Investigaciones Clínicas Norberto Quirno “CEMIC”, Ciudad Autónoma de Buenos Aires C1425ASG, Argentina; eridruejo@cemic.edu.ar; 4 Hepatology and Liver Transplant Unit, Hospital Universitario Austral Pilar, Provincia de Buenos Aires B1629AHJ, Argentina; 5 Latin American Liver Research, Educational and Awareness Network (LALREAN) Pilar, Provincia de Buenos Aires B1629AHJ, Argentina

**Keywords:** chronic kidney disease, hepatocellular carcinoma, hepatitis B virus, hepatitis C virus, liver transplant

## Abstract

**Simple Summary:**

Chronic kidney disease is a major public health issue globally and the risk of hepatocellular cancer appears greater in patients with chronic kidney disease compared with the general population. Non-alcoholic fatty liver disease is a liver disorder ranging from simple fatty infiltration to advanced fibrosis plus inflammation; it plays a role in developing liver-related and extra liver-related diseases including HCC and CKD, respectively. Approximately 90% of HCCs are associated with a known underlying etiology; viral hepatitis is a well-known cause of HCC, particularly in CKD population. Antiviral therapy of HBV and HCV is important in the management of HCC in CKD patients. Therapy of HCC in CKD patients includes liver transplant (in selected patients), local approach (surgery or interventional radiology), and tyrosine kinase inhibitors (advanced HCC).

**Abstract:**

Chronic kidney disease is a major public health issue globally and the risk of cancer (including HCC) is greater in patients on long-term dialysis and kidney transplant compared with the general population. According to an international study on 831,804 patients on long-term dialysis, the standardized incidence ratio for liver cancer was 1.2 (95% CI, 1.0–1.4) and 1.5 (95% CI, 1.3–1.7) in European and USA cohorts, respectively. It appears that important predictors of HCC in dialysis population are hepatotropic viruses (HBV and HCV) and cirrhosis. 1-, 3-, and 5-year survival rates are lower in HCC patients on long-term dialysis than those with HCC and intact kidneys. NAFLD is a metabolic disease with increasing prevalence worldwide and recent evidence shows that it is an important cause of liver-related and extra liver-related diseases (including HCC and CKD, respectively). Some longitudinal studies have shown that patients with chronic hepatitis B are aging and the frequency of comorbidities (such as HCC and CKD) is increasing over time in these patients; it has been suggested to connect these patients to an appropriate care earlier. Antiviral therapy of HBV and HCV plays a pivotal role in the management of HCC in CKD and some combinations of DAAs (elbasvir/grazoprevir, glecaprevir/pibrentasvir, sofosbuvir-based regimens) are now available for HCV positive patients and advanced chronic kidney disease. The interventional management of HCC includes liver resection. Some ablative techniques have been suggested for HCC in CKD patients who are not appropriate candidates to surgery. Transcatheter arterial chemoembolization has been proposed for HCC in patients who are not candidates to liver surgery due to comorbidities. The gold standard for early-stage HCC in patients with chronic liver disease and/or cirrhosis is still liver transplant.

## 1. Introduction

Solid evidence suggests an increased risk of cancer after kidney transplant; on the contrary, the risk of cancer in patients receiving dialysis is more controversial. The majority of data suggest that patients on maintenance dialysis remain at increased risk of malignancy due to various reasons including abnormalities in immune response, nutritional changes, prior treatment with immunosuppressive agents, and greater frequency of chronic infections. In fact, hemodialysis environment supports the acquisition of blood-borne infections such as hepatotropic viruses, which may lead to development of chronic liver disease and liver cancer.

Hepatocellular carcinoma is the most frequent primary cancer of liver and approximately 90% of HCCs are related to a clear etiology (i.e., viral hepatitis, alcohol intake, or aflatoxin exposure). Chronic kidney disease has been recognized as a leading public health issue worldwide (Table 1); the global prevalence of CKD is 13.4% (95% CI, 11.7–15.1%) and the number of patients with end-stage renal disease who require kidney replacement therapy is estimated between 4.902 and 7.083 million [1,2]. The relationship between HCC and CKD has been investigated by various authors and the outcomes of patients with HCC and CKD are controversial even though both can be endemic in various countries.

The aim of the current narrative review is to summarize the most recent acquisitions concerning the epidemiology and management of HCC in patients with CKD.

## 2. Epidemiology of Cancer in Dialysis Population vs. Controls

Some data have been accumulated in the last decade on the epidemiology and risk factors for HCC in patients with CKD. Novel information has been given by a Korean nationwide study (The Korean National Health Insurance Database) that included 48,315 dialysis patients and 48,315 controls (selected via propensity score matching) [3]. The overall cancer risk was greater in dialysis population than among controls (adjusted HR = 1.71; 95% CI, 1.62–1.81). Compared with the general population, kidney cancer (IRR = 6.75; 95% CI, 4.85–9.6, *p* < 0.001), followed by upper urinary tract cancer (IRR = 4.0; 95% CI, 2.23–7.54, *p* < 0.001) and skin cancer (IRR = 3.38; 95% CI, 2.35–4.93, *p* < 0.001) were more common in dialysis patients. The rate of liver/biliary cancer was greater in dialysis population (IRR = 1.61, 95% CI, 1.41–1.84, *p* < 0.0001).

A collaborative study which assembled a cohort of 831,804 patients who underwent regular dialysis (1980–1994) for end stage kidney disease in Europe, USA, Australia, and New Zealand [4] has been published. The frequency of cancer in the respective background populations was assessed. The investigators observed that 25,044 of 831,804 patients developed cancer in comparison with an expected number of 21,185 during an average follow-up of 2.5 years, the SIR was 1.18 (95% CI, 1.17–1.20). The rate of liver cancer was greater in ESRD than controls in European and USA cohort, SIR = 1.2; 95% CI, 1.0–1.4 and SIR = 1.5, 95% CI, 1.3–1.7, respectively. The SIR for liver cancer was 1.5 (0.5–4.6) in the cohort from Australia and New Zealand. 

Another piece of evidence on this topic has been offered by a survey on 92,348 chronic dialysis patients retrieved from the National Health Insurance Research Database (NHIRD) during 1997–2008 [5]. Background cancer incidence rates for the general population were calculated from the cancer registry given from the Department of Health. The SIR of overall cancer in chronic dialysis patients was greater than in the general population (SIR = 1.4, 95% CI, 1.3–1.4). The SIR of liver cancer was 1.4 (95% CI, 1.2–1.5) indicating that liver cancer was more common than among healthy counterparts [5].

The cancer risk associated with dialysis has been addressed in a population-based cohort study of 28,855 patients (Australia and New Zealand Dialysis and Transplant Registry, ANZDATA) with end-stage kidney disease; 24,926 patients (14,144 men) underwent dialysis with a mean follow-up of 2.7 ± 2.5 years. The SIR of 1.35 (95% CI, 1.27–1.45) was greater than that for the before dialysis period (*p* = 0.02) [6].

According to the claims data of the Bureau National Health Insurance of Taiwan, 38,714 patients with ESRD were enrolled for the study, and a database of 1 million individuals who were randomly selected and matched for some background and clinical parameters was adopted as control group (*n* = 38,714) [7]. No difference occurred in the incidence of HCC between ESRD patients and controls, 2.03 per 1000 person-years vs. 2.10 per 1000 person-years, RR (rate ratio) = 0.947 (95% CI, 0.792–1.132, NS). After stratification by age and gender and being adjusted for age, gender, DM, arterial hypertension, heart failure, and gout, no difference in the incidence for the development of HCC between ESRD and non-ESRD patients was noted. The conclusion of the investigators was that, after matching for hepatitis and liver cirrhosis, there is no greater incidence of HCC in patients with end-stage renal disease.

## 3. Risk Factors for HCC in Dialysis Population

Henderson and colleagues evaluated the predictors of HCC in dialysis patients with chronic HCV [8]. Data were extracted from the USRDS (United States Renal Database System) using ICD-9 codes. Among the 32,806 patients with HCV infection, 262 had HCC. The incidence of HCC in the sample on dialysis during the study period was 0.8%. The authors found that HCC was more common in patients with cirrhosis (OR = 11.7, 95% CI, 8.89–15.5). The role of HBV, drug abuse, and HIV in this subset of patients remained unclear. The correlational matrix revealed a significant relationship between cirrhosis and alcohol abuse (*r* = 0.35, *p* < 0.001). The study confirmed the association between cirrhosis and HCC, cirrhosis increases the risk for HCC by multiple mechanisms.

Another important risk factor for HCC in dialysis patients remains hepatitis B; as suggested by a retrospective survey carried out in Taiwan, an endemic HBV area. Tung and coworkers found 13 patients receiving regular hemodialysis and diagnosed with HCC over the period 1991–1997. There were six patients with HBV-related and seven with HCV-related HCC. The investigators did not find difference between the two groups with respect to aminotransferase levels, bilirubin, alpha-fetoprotein, and mean time on dialysis (29.7 ± 22.1 vs. 87.9 ± 79.9 months, NS) [9].

The role of viral hepatitis status in the development of HCC has been noted by Yu and coworkers in a cohort of kidney transplant recipients [10]. Individuals with kidney transplant were identified from the catastrophic illness registry of National Health Insurance Research Database (NHIRD) during the period 2000–2009 [10]. Renal transplant recipients with HBV alone, HCV alone, and both with HBV and HCV infection, respectively, showed a greater hazard ratio (aHR = 9.84, 95% CI, 4.6–21; aHR = 4.4, 95% CI, 1.8–10.5; aHR = 4.63, 95% CI, 1.06–20.2) of HCC in comparison with those who had neither HBV or HCV infection. aHRs were adjusted for several background and clinical parameters.

## 4. Survival of HCC: CKD vs. Intact Kidneys

The survival of chronic kidney disease patients with HCC remains an area of active research. Hepatocellular carcinoma is the most common primary liver cancer and is currently recognized the fourth leading cause of cancer mortality worldwide, accounting for nearly 700,000 deaths/year [11]. Some data have been published on the outcomes of HCC in CKD patients [12,13]. Lee and coworkers [13] conducted a retrospective observational study in northern Taiwan; 440 patients were referred between 2000 and 2002 for management of HCC and categorized according to their CKD stage. In a multivariate analysis (Cox regression model), CKD stage (OR, 1.98, 95% CI, 1.01–3.9, *p* = 0.046), liver cirrhosis stage (OR, 3.57, 95% CI, 1.59–8.0, *p* = 0.002), and serum albumin concentration (OR, 0.657, 95% CI, 0.49–0.87, *p* = 0.005) were independent predictors for death. Patients with stage 4 and 5 CKD showed lower cumulative survival than those with stages 1 and 2 CKD (log-rank test, *χ*^2^ = 11.76, *p* = 0.003).

Another survey from Taiwan enrolled 1298 patients with HCC, of whom 172 (13.2%) were receiving regular hemodialysis (serum creatinine, 8.4 ± 2.7 mg/dL) and 1126 (serum creatinine, 0.9 ± 0.2 mg/dL) were not [14]. Serum alkaline phosphatase levels were greater in the hemodialysis than non-hemodialysis group, 162.8 ± 141.1 vs. 124.6 ± 102.5 u/L, *p* < 0.001. Kaplan–Meier analysis demonstrated that cumulative survival was lower in HCC patients on regular hemodialysis (*p* = 0.004). The 1-, 3-, and 5- year survival rates were 78%, 67.9%, and 54.4% for patients with HCC on hemodialysis vs. 88.3%, 74.5% and 64.8% for those without hemodialysis. According to the multivariate Cox regression model, hemodialysis (*p* = 0.001), older age (*p* < 0.001), and advanced tumor stages (*p* < 0.001) were independent predictors for mortality. The death risk of patients having HCC who received hemodialysis was 2.036 greater than in those HCC patients who did not receive HD.

Lee and colleagues [15] made a retrospective analysis and retrieved a total of 2502 patients with HCC, including 30 patients on dialysis and 90 controls (matched for age, gender, and treatment). No difference in survival between dialysis, non-dialysis patients (*p* = 0.684), and matched controls (*p* = 0.373) was found.

Between 2002 and 2016, 3690 patients with new diagnosis of HCC were admitted to Taipei General Hospital [11]. 1000 patients had kidney insufficiency (serum creatinine, 2.0 ± 1.9 mg/dL) and 2690 (serum creatinine, 0.9 ± 0.2 mg/dL) were without it. During a mean follow-up period of 37 months, patients with kidney insufficiency showed decreased survival in comparison with those patients without kidney insufficiency, the 1- and 3- year survival rates of patients with renal insufficiency were 60% and 39% vs. 69% and 50% in those without RI, respectively (*p* < 0.001). Multivariate survival analysis showed that age older than 65 years (HR, 1.160, *p* < 0.001), male gender (HR, 1.207, *p* < 0.001), eGFR <60 mL/min/1.73 m^2^ (HR, 1.234, *p* < 0.001), multiple tumors (HR, 1.136, *p* = 0.003), and vascular invasion (HR, 2.486 *p* < 0.001), among others, were significantly linked with decreased survival.

Toyoda and coworkers [16] conducted a survey on 108 patients on dialysis (diagnosed as having naïve, non-recurrent HCC between 1998 and 2015) who were compared with 526 controls without dialysis with naïve HCC followed at Ogaki Municipal Hospital. According to multivariate analysis, factors associated with survival in HCC population were age (HR, 1.02, 95% CI 1.01–1.03, *p* = 0.0041), serum albumin (HR, 0.46, 95% CI, 0.37–0.58, *p* < 0.0001), serum bilirubin (HR, 1.4, 95% CI, 1.21–1.61, *p* < 0.0001) and dialysis (HR, 1.66, 95% CI, 1.21–2.24, *p* = 0.002). The 1-, 3- and 5-year survival rates of patients on dialysis were 79.5%, 56.3%, and 38.3%, respectively, which were lower than those of non-dialysis controls. 87.6%, 66.5%, and 52.7%, respectively (*p* = 0.0026). The results were confirmed after propensity score matching (*p* = 0.0014). The conclusion of the authors was that HCC was more advanced at diagnosis in patients on dialysis than in non-dialysis controls where diagnosis of HCC was made during surveillance at liver centers. The survival rates after diagnosis were reduced in dialysis population.

In the survey of Hwang and colleagues [7], the Cox proportional hazard methods were applied to evaluate the risk factors associated with long-term mortality in patients with HCC (*n* = 493 patients; end-stage renal disease (*n* = 214) and non- end-stage renal disease (*n* = 279)). Multivariate analysis was conducted and adjustment was made for age, gender, diabetes mellitus, arterial hypertension, gout, and congestive heart failure. Risk factors independently and significantly associated with long-term mortality in patients with HCC were diabetes (aHR, 1.55, 95% CI, 1.13–2, 11, *p* = 0.005) and end-stage renal disease (aHR, 1.61, 95% CI, 1.19–2.189, *p* = 0.002).

A large, population-based prospective cohort was conducted in Taiwan [17]. A total of 123,717 adults were recruited (5150 individuals had dialysis independent CKD and 118,567 patients had normal kidney function). Patients were followed during a median time of 7.06 years; 2710 deaths occurred. Patients with CKD had a higher risk for overall cancer mortality, adjusted HR, 1.20 (95% CI, 1.02 to 1.42). CKD was significantly associated with mortality caused by liver cancer, aHR, 1.74 (95% CI, 1.24 to 2.44). Deaths from liver cancer, kidney cancer, and urinary tract cancer increased incrementally with the severity of kidney impairment. This was the first large study that reported a greater death risk for liver cancer in patients with CKD not yet on dialysis.

## 5. HCC and CKD in Chronic HB Patients: Experience during 2000–2015

According to recent longitudinal studies [18,19,20], the population of patients with chronic hepatitis B is aging and the frequency of some liver (HCC) and non-liver (CKD) complications is increasing along the time. A large multicenter retrospective, observational study has been recently published which enrolled consecutive CHB patients in northern California [18]. A total of 2734 adult American CHB patients were retrieved from a university medical center and several community primary care clinics. Individual medical records were reviewed and confirmed 2734 adult (>18 years) patients with CHB (positive HBsAg or HBV DNA). Mean age increased consistently (43 ± 13.4 years during 2000–2005 to 49.1 ± 14.4, *p* < 0.001); the authors found increasing trends over time in various liver and non-liver comorbidities, in both men and women, and among both treated and untreated patients. The proportion of chronic hepatitis B patients with HCC significantly increased between 2000 and 2015, 4.9% (2000–2005), 4.8% (2005–2010), and 9.1% (2011–2015) (_Χ_^2^ test *p* < 0.001). In the study, men had more advanced liver disease (such as HCC) than women. The proportion of CHB patients having chronic kidney disease increased significantly between 2000 and 2015, 4.41% (2000–2005), 9.7% (2005–2010), and 19.7% (2011.2015), (_Χ_^2^ test *p* < 0.001). The conclusion was that over the last 15 years, more patients with chronic hepatitis B are presenting with advanced liver disease (including HCC) or chronic kidney disease without prior treatment. A delayed referral to subspecialty or university clinics but also a delay in linkage to care at a community primary care level exists. Additional efforts are required to make diagnosis of HBV earlier and to link these patients to appropriate care.

Some investigators evaluated the prevalence and incidence of non-liver comorbidities in patients with chronic hepatitis B having continuous coverage 6 months prior to and after the first diagnosis of chronic hepatitis B; these patients were matched with individuals without chronic hepatitis B [19]. The study population included 44,026 CHB cases and 121,568 matched controls who were identified by insurance claim databases. Mean age increased over time: it was 48.1 ± 11.9 years (2006) and increased to 51.8 ± 12.4 years (2015) for the commercial/Medicare (*p* < 0.001). Mean age ranged from 44.1 ± 11.1 years (2006) to 50.2 ± 10.2 years (2015) for Medicaid (*p* < 0.001). The commercial/Medicare prevalence rate of CKD (2006) was 36.1/1000 in CHB patients and 10.2/11,000 in controls, and it increased to 97.6% and 38.8% in 2015, respectively. The strongest predictors for CKD were DM (HR, 2.47 95% CI, 2.32; 2.62), arterial hypertension (HR, 3.29, 95% CI, 3.09; 3.63) and cardiovascular disease (HR, 2.61; 2.44; 2.78) (all *p* < 0.0001). The take-home message of the authors was that we need to link CHB patients to care at a younger age when they are in a healthier state.

## 6. HCC, NAFLD and Chronic Kidney Disease

**Figure 1:**
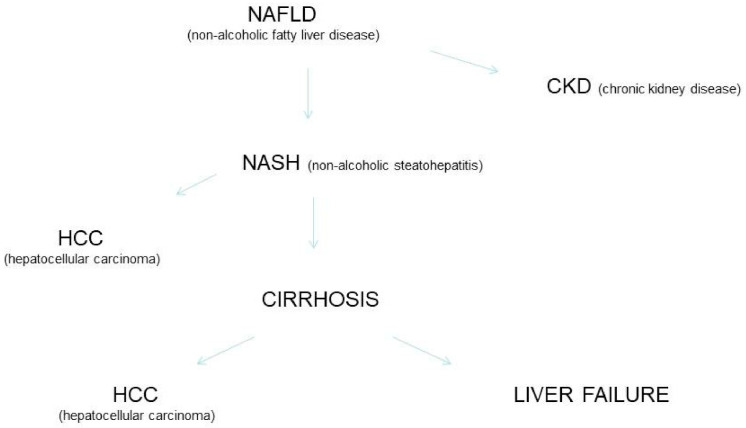
NAFLD, progression of liver damage and extra-hepatic complications.

Non-alcoholic fatty liver disease is a systemic disorder with complex pathogenesis and various clinical manifestations. NAFLD has been accepted a major cause of advanced fibrosis, cirrhosis, liver failure, and HCC. More recently, NAFLD has been linked with extra-hepatic manifestations such as diabetes mellitus, cardiovascular and chronic kidney disease. It has been calculated that around 20% of HCC cases in the USA are currently related to NAFLD. The risk of developing HCC among NAFLD patients is increased by various risk factors such as metabolic syndrome, ethnicity, and hepatic siderosis. The cumulative incidence of HCC in patients with NASH-related cirrhosis is great and ranges from 2.4% over 7 years to 12.8% over 3 years. In a subset of patients with NASH, HCC can develop de novo in the absence of cirrhosis. The course of liver disease progression in NAFLD is unclear and a subset of these patients show progressive liver disease leading to NASH, cirrhosis, and HCC (Figure 1). Numerous investigators suggested that NAFLD is on trajectory to become the most frequent chronic liver disease requiring liver or liver-kidney transplant [21,22,23].

According to a systematic review with meta-analysis of clinical observational studies (*n* = 33, 63,902 unique patients) NAFLD was linked with an increased risk of prevalent (OR, 2.12, 95% CI, 1.69–2.66) and incident (HR, 1.79 (95% CI, 1.65–1.95) CKD [24]. The pooled estimate of the risk of prevalent CKD is greater in patients with advanced fibrosis (F3) in comparison with no advanced fibrosis (F0-F2) in patients with biopsy proven non-cirrhotic NAFLD (*n* = 8 reports, *n* = 969 unique patients) (OR 5.2, 95% CI, 3.14–8.61). The overall estimate of the risk of incident CKD is greater in patients with advanced fibrosis vs. no-advanced fibrosis in patients with biopsy proven non-cirrhotic NAFLD (*n* = 6 reports, *n* = 429 studies), HR, 3.29 (95% CI, 2.3–4.71). The conclusion was that the association occurred in cross-sectional and longitudinal studies and after taking various confounders into account. Additionally, the association was present across various criteria for NAFLD diagnosis (histology, imaging, and biochemical tests).

An updated meta-analysis recently reported that NAFLD was associated with a 40% increase in the risk of incident CKD > 3, HR, 1.37 (95% CI, 1.2–1.63) (median follow-up, 5.2 years) [25].

## 7. Antiviral Therapy for HCC in Chronic Kidney Disease (HCV)

Treatment of HCV plays a crucial role in the management of HCC in CKD patients as chronic HCV infection is an important cause of HCC in dialysis population and after kidney transplant. Until a few years ago, the standard of care was pegylated interferon in combination with ribavirin for 24 or 48 weeks. This combination gave limited cure rates (50–60%) and was poorly tolerated by patients. The efficacy and tolerability of IFN-based therapy was much lower in patients with CKD compared with individuals with intact kidneys [26].

The understanding of the HCV genome and proteins has made possible the development of direct-acting antiviral agents (DAAs) that are molecules targeting specific non-structural proteins of the virus in order to interrupt viral replication and infection of HCV. DAA-based therapies for HCV first became available in 2011, now include numerous all-oral, interferon-free, and ribavirin-free regimens provided with great efficacy (>95%) and satisfactory safety [27]. Four classes of DAAs exist, which are defined by their mechanism of action and therapeutic target. The advent of direct-acting antiviral agents has dramatically changed the treatment of HCV not only in the general population, but also in ‘special populations’ (including CKD population). A few combinations of DAAs have been currently recommended for antiviral treatment of HCV in patients with CKD. As listed in Table 2, elbasvir/grazoprevir, glecaprevir/pibrentasvir, and sofosbuvir-containing regimens have been recommended for the treatment of patients with CKD stage 1–5 and no dose adjustment is required.

The C-SURFER trial [28,29] assessed the efficacy and safety of elbasvir (50 mg)/grazoprevir (100 mg) vs. placebo in patients with HCV genotype 1 and CKD stage 4/5. In the initial study eligible patients were randomized to immediate or deferred treatment; patients randomized to deferred treatment were later treated with elbasvir/grazoprevir. Elbasvir and grazoprevir are metabolized at liver level and their clearance by kidneys is extremely small. Based on the C-SURFER trial data, daily fixed-dose elbasvir/grazoprevir is recommended for the treatment of HCV genotype 1 and 4 in patients with chronic kidney disease stage 4/5.

The EXPEDITION-4 is an open label study enrolling treatment-naïve and experienced patients with CKD stage 4/5; patients on maintenance dialysis were also included. HCV-infected individuals with genotype 1, 2, 3, 4, 5, and 6 were treated; experienced patients had previously received antiviral therapy with IFN-based (conventional or pegylated interferon +/− ribavirin) or SOF-based therapy (sofosbuvir plus ribavirin +/− pegylated interferon) [30]. The EXPEDITION-4 trial highlights the efficacy and safety of the daily fixed-dose combination of glecaprevir (300 mg)/pibrentasvir (120 mg) for the antiviral treatment of HCV in the CKD population, including those with 4–5 CKD stage. 

Sofosbuvir, a non-structural NS5B polymerase inhibitor, has been approved in 2013 and is now the backbone of many DAA treatment regimens. SOF has a large excretion by kidneys and has been initially licensed only for patients with eGFR > 30 mL/min/1.73 m^2^. Sofosbuvir usually undergoes intracellular metabolism in the liver and the most important circulating metabolite of sofosbuvir (GS-331007) is mostly cleared by kidneys and achieves up to 456% increase in AUC in patients with creatinine clearance < 30 mL/min compared with those having intact kidneys [31,32]. Despite these pharmacokinetic data, numerous ‘real life’ studies have suggested high efficacy (SVR rate > 90%) and safety of SOF-based regimens including sofosbuvir/ledipasvir [33] and sofosbuvir/velpatasvir [34] in HCV-infected patients with an eGFR < 30 mL/min. In November 2019, the FDA gave license to use SOF-containing regimens in CKD patients, including those with eGFR < 30 mL/min and maintenance dialysis.

Overall, the data reported above indicate that HCV infection is a ‘curable’ disease even in patients with CKD and stage 4/5 or on dialysis; DAAs give SVR rates greater > 90% and good safety in these patients.

## 8. Antiviral Therapy for HCC in Chronic Kidney Disease (HBV)

HBV infection remains a significant agent for the development of HCC in patients with CKD. The first –line nucleos(t)ide analogues for the treatment of patients with hepatitis B are currently the following: entecavir (ETV), tenofovir disoproxil fumarate (TDF), and tenofovir alafenamide (TAF). Reports on the use of NAs for hemodialysis patients are quite poor and these mostly regard lamivudine [35,36].

Ridruejo and coworkers administered entecavir to eight patients with advanced CKD (one with CKD stage 4 and seven on maintenance hemodialysis) [37]. The mean ETV treatment duration was 1.91 months (range, 0.68–3.27). HBV DNA became undetectable in six patients, HBV DNA reduction occurred with a range between 0.78 and 2.41 logs in the other three patients. In their multicenter retrospective study in Japan, the NORTE study group enrolled 40 patients with CKD- 25 had CKD stage 3, 5 stage 4/5 and 10 on maintenance hemodialysis. All patients received ETV monotherapy for at least 1 year; the rate of HBV DNA clearance from serum was 96.3% (26/27) three years after initiation of mono-therapy with ETV. The frequency of ALT normalization at 36 months was 88.9% (24/27), the rate of HBeAg disappearance at 12 months was 42.8% (3/7). Viral breakthrough occurred in two (5%) patients [38]. The entecavir dose in patients with kidney impairment is reported in Table 3.

## 9. HCC, Chronic Kidney Disease and Tyrosine Kinase Inhibitors

The prognosis of patients with HCC at advanced stage remains poor despite improvements in local approaches (surgery and interventional radiology) or liver transplantation (in selected candidates) for early or intermediate stages of HCC. The first drug approved for advanced HCC was the tyrosine kinase inhibitor sorafenib: it has been used a first-line treatment for HCC [39]. The efficacy and safety of sorafenib has been successfully tested in various phase III and real-life studies including CKD population. Data on pharmacokinetics showed no difference in plasma level of the drug between plasma with intact kidneys and those with kidney impairment. After 10 years, lenvatinib which is another tyrosine kinase inhibitor, has been licensed as first-line treatment of HCC. Studies are in progress to give support to clinicians in the choice between sorafenib and lenvatinib.

## 10. HCC, Chronic Kidney Disease and Interventional Management (1)

The management of HCC is more difficult among patients with CKD and when the cancer is at an advanced stage. Some interventional approaches (including hepatic resection) are available when diagnosis of HCC is performed at an early stage. The interventional approaches are safer than in the past due to many advances in preoperative management, diagnostic imaging, patient selection, surgical techniques, and postoperative care. Liver resection is now a safe surgical procedure provided with a low mortality rate; the risk of operative complications is increased in CKD population as several non-liver comorbidities typically affect patients with CKD [40]. A cohort study based on the Taiwan’s National Health Institute Research Database enrolled 149 patients with HCC and uremia who underwent hepatic resection between 1996 and 2008; 596 non-uremic patients with HCC were controls and received hepatic resection during the same time period. The two groups were matched for various parameters and no difference between HCC with uremia or not in survival outcomes, regardless of extent of hepatic resection, occurred. The risk of post-operative infection-related complications requiring intervention (4.03% vs. 1.17%, *p* = 0.0175) and life-threatening heart associated complications (2.01% vs. 0.17%, *p* < 0.005) was greater in HCC patients with uremia than among non-uremic HCC individuals [41].

Yeh and colleagues retrospectively evaluated the outcome of 26 patients with HCC and end-stage kidney disease who underwent liver resection from 1982 to 2001; the outcomes of 1198 HCC patients without end-stage kidney disease were adopted for comparison. They found that overall (*p* = 0.70) and disease-free survival rates (*p* = 0.61) were not different between the two groups. The conclusion of the authors was that comparable survival to non-uremic patients with HCC can be achieved in selected HCC patients with ESRD undergoing liver resection [42].

Some ablative techniques (microwave or radiofrequency ablation, among others) have been introduced for HCC in CKD patients who are not candidates for surgery. RFA is the ablative technology usually described in CKD patients with HCC—it has been demonstrated to be effective for HCC no more than 5 cm in size. An important survey on the safety and efficacy of RFA in patients on hemodialysis has been carried out by Sato and coworkers [43]. They adopted the Japanese Diagnosis Procedure Combination database and enrolled 437 patients on regular hemodialysis and 1345 matched non dialyzed patients. For each patient, up to four non-dialyzed patients were randomly selected by a matched-pair sampling method based on patient age, gender, treatment hospital, and treatment year. In-hospital mortality was consistently greater in dialysis than among non-dialysis patients (1.1% vs. 0.15%, *p* < 0.001). Hemorrhagic complications were significantly more common in dialysis than non-dialysis patients, 3.4% vs. 0.87%, respectively, *p* < 0.001). The major risk of RFA in dialysis population was given by hemorrhagic complications due to platelet dysfunction and heparin use.

## 11. HCC, Chronic Kidney Disease and Interventional Management (2)

An additional interventional procedure is TACE (transcatheter arterial chemoembolization) that has been proposed for unresectable HCC in patients who are not candidates to liver surgery due to general comorbidities. TACE requires ultra-selective liver angiography resulting in tumor necrosis by embolization of the artery supplying the tumor. Lin et al. evaluated 132 patients who received at least one procedure of transarterial therapy for initial diagnosis of HCC at Mackay Memorial Hospital, Taipei (2014–2016) [44]. Among them, 36 patients had CKD and underwent 58 TACE sessions. Estimated GFR decreased 13.7% (*p* < 0.01) and 2.2% (NS) from baseline after therapy in the CKD and non-CKD group, respectively. Patient survival (from initial diagnosis of HCC to patient death) was lower in the CKD than in non-CKD group (10.9 ± 8.5 vs. 23.5 ± 16.3 months, *p* < 0.01) and the most common cause of death was deterioration of HCC, followed by sepsis and decompensated cirrhosis. No difference in the causes of mortality in both the groups was reported. The authors gave emphasis to the acute deterioration in kidney function (due to administration of contrast media) post-TACE. Various causes are associated with the occurrence of post-TACE contrast induced nephropathy (CIN); in order to prevent CIN, peri-procedural hydration and/or oral acetylcysteine have been suggested.

## 12. HCC, Chronic Kidney Disease and Interventional Management (3)

Liver transplantation is currently the better approach for early stage HCC in patients with chronic liver disease and/or cirrhosis. LT aims to treat the neoplastic disorder and the underlying liver disease; HCC patients have reduced time on the waiting list due to the occurrence of drop-outs. The MELD scoring system has been adopted since 2002 to assess the mortality risk of candidates while on the waiting list for liver transplant. The calculation of MELD score is performed by serum creatinine, bilirubin levels, and international normalized ratio [45,46]. Greater scores suggest advanced stages of chronic liver disease, MELD score is currently adopted by the United Network for Organ Sharing (UNOS) and Eurotransplant for better allocation of liver transplant instead of obsolete Child-Pugh score.

An appropriate evaluation of kidney function plays a pivotal role for identification of candidates for combined transplant (simultaneous kidney/liver transplant). The progressive implementation of the MELD scoring system has made more frequent the CLKT strategy. The comparison of outcomes and post-transplant kidney function between patients who underwent kidney/liver transplant and liver transplant alone was made using eGFR stratification. Tinti and coworkers [47] analyzed the UK National Transplant Database (NHSBT) and evaluated 6035 patients receiving an LTA (*n* = 5912; 98%) vs. CLKT (*n* = 123; 2%); analysis was made after stratification by KDIGO stages of eGFR at transplant. No difference in patient and graft survival between LTA and CLKT patients in various eGFR group/strata occurred (NS). There were 377 patients on maintenance dialysis at LT- 305 (81%) and 72 (19%) underwent LTA and CLKT, respectively. Patients who underwent CLKT had better patient (*p* = 0.03) and graft (*p* = 0.01) survival than those who received LTA. The conclusion was that the advantage of CLKT is apparent only in the subset of liver transplant candidates on kidney replacement therapy at the time of LT.

## 13. Conclusions

Patients with chronic kidney disease such as patients on maintenance dialysis and kidney transplant recipients are at risk for development of cancer including hepatocellular carcinoma. Liver cancer plays a detrimental role on survival in chronic kidney disease patients, dialysis—dependent or not. Non-alcoholic fatty liver disease, once believed to be a benign condition, can progress to cirrhosis and hepatocellular carcinoma. The non-interventional management of HCC includes antiviral therapy for HBV and HCV, which are important agents of HCC in chronic kidney disease population, and some antiviral drugs or drug combinations have been recently approved in advanced CKD. The interventional management of HCC is improved due to recent advances in patient selection, diagnostic imaging, preoperative management, and postoperative care. Liver transplant for early stage HCV is effective even in patients with advanced CKD; combined liver/kidney transplant is currently recommended in selected individuals.

## Figures and Tables

**Table 1:** Stages of chronic kidney disease and eGFR levels.

CKD Stage		eGFR Level
1	Kidney damage with normal or increased GFR	≥90 mL/min
2	Kidney damage with mild decrease of GFR	60–89 mL/min
3	Moderate reduction of GFR	30–59 mL/min
4	Severe reduction of GFR	15–29 mL/min
5	Kidney failure	<15 mL/min or on dialysis

**Table 2:** DAAs for treatment of HCV-associated liver disease in CKD (according to AASLD/IDSA guidelines).

Drug	Activity	Dose	Reference
Elbasvir/Grazoprevir	NS5A inhibitor NS3/4A protease inhibitor	Elbasvir 50 mg Grazoprevir 100 mg Daily	ClinicalTrials.gov number, NCT02092350 C-SURFER Trial [21]
Glecaprevir/Pibrentasvir	NS3/4A protease inhibitor NS5A inhibitor	Glecaprevir 100 mg Pibrentasvir 40 mg three times daily	ClinicalTrials.gov number, NCT02651194 EXPEDITION-4 trial [23]
Sofosbuvir/Ledipasvir	NS5B polymerase inhibitor NS5A inhibitor	400 mg Sofosbuvir 90 mg Ledipasvir Daily	ClinicalTrials.gov number, NCT01958281 [26]
Sofosbuvir/velpatasvir	NS5B polymerase inhibitor NS5A inhibitor	Sofosbuvir 400 mg Velpatasvir 100 mg daily	ClinicalTrials.gov number, NCT03036852 [27]

**Table 3:** Baraclude and tenofovir for treatment of HBV-associated liver disease in CKD. * compensated liver disease.

Creatinine Clearance	Entecavir *	Tenofovir Disoproxil *
>50 mL/min/1.73 m^2^	0.5 mg per day	245 mg per day
30–49 mL/min/1.73 m^2^	0.25 mg per day	245 mg/48 h
10–29 mL/min/1.73 m^2^	0.15 mg per day	245 mg x2/week
<10 mL/min/1.73 m^2^ (including patients on dialysis)	0.05 mg per day	245 mg/week

## Abbreviations

AASLD	American Association for the Study of Liver Diseases
AH	Arterial Hypertension
CI	Confidence intervals
CIN	Contrast induced nephropathy
CKD	Chronic kidney disease
DAA	Direct-acting antiviral agent
DM	Diabetes mellitus
eGFR	Estimated glomerular filtration rate
ESRD	End-stage renal disease
GN	Glomerulonephritis
HBV	Hepatitis B virus
HCC	Hepatitis C virus
HCV	Hepatocellular carcinoma
HD	Hemodialysis
IDSA	Infectious Disease Society of America
IFN	Interferon
ITT	Incidence rate ratio
LT	Liver transplant
NA	Not available
OR	Odds ratio
PegIFN	Pegylated interferon
RBV	Ribavirin
RR	Relative risk
RT	Renal transplant
SOF	Sofosbuvir
SVR	Sustained Virological Response
W12	12 weeks after antiviral therapy ended
